# DAPK1 Promotes Extrasynaptic GluN2B Phosphorylation and Striatal Spine Instability in the YAC128 Mouse Model of Huntington Disease

**DOI:** 10.3389/fncel.2020.590569

**Published:** 2020-11-05

**Authors:** Mandi E. Schmidt, Nicholas S. Caron, Amirah E. Aly, Fanny L. Lemarié, Louisa Dal Cengio, Yun Ko, Nikola Lazic, Lisa Anderson, Betty Nguyen, Lynn A. Raymond, Michael R. Hayden

**Affiliations:** ^1^Centre for Molecular Medicine and Therapeutics, BC Children’s Hospital Research Institute, University of British Columbia, Vancouver, BC, Canada; ^2^Department of Psychiatry and Djavad Mowafaghian Centre for Brain Health, University of British Columbia, Vancouver, BC, Canada

**Keywords:** DAPK1, synaptic, GluN2B, pS1303, Huntington disease, YAC128, medium spiny neuron, NMDA receptor

## Abstract

Huntington disease (HD) is a devastating neurodegenerative disorder caused by a CAG repeat expansion in the huntingtin gene. Disrupted cortico-striatal transmission is an early event that contributes to neuronal spine and synapse dysfunction primarily in striatal medium spiny neurons, the most vulnerable cell type in the disease, but also in neurons of other brain regions including the cortex. Although striatal and cortical neurons eventually degenerate, these synaptic and circuit changes may underlie some of the earliest motor, cognitive, and psychiatric symptoms. Moreover, synaptic dysfunction and spine loss are hypothesized to be therapeutically reversible before neuronal death occurs, and restoration of normal synaptic function may delay neurodegeneration. One of the earliest synaptic alterations to occur in HD mouse models is enhanced striatal extrasynaptic NMDA receptor expression and activity. This activity is mediated primarily through GluN2B subunit-containing receptors and is associated with increased activation of cell death pathways, inhibition of survival signaling, and greater susceptibility to excitotoxicity. Death-associated protein kinase 1 (DAPK1) is a pro-apoptotic kinase highly expressed in neurons during development. In the adult brain, DAPK1 becomes re-activated and recruited to extrasynaptic NMDAR complexes during neuronal death, where it phosphorylates GluN2B at S1303, amplifying toxic receptor function. Approaches to reduce DAPK1 activity have demonstrated benefit in animal models of stroke, Alzheimer’s disease, Parkinson’s disease, and chronic stress, indicating that DAPK1 may be a novel target for neuroprotection. Here, we demonstrate that dysregulation of DAPK1 occurs early in the YAC128 HD mouse model, and contributes to elevated extrasynaptic GluN2B S1303 phosphorylation. Inhibition of DAPK1 normalizes extrasynaptic GluN2B phosphorylation and surface expression, and completely prevents YAC128 striatal spine loss in cortico-striatal co-culture, thus validating DAPK1 as a potential target for synaptic protection in HD and warranting further development of DAPK1-targeted therapies for neurodegeneration.

## Introduction

Huntington disease (HD) is a debilitating and fatal neurodegenerative disorder caused by a CAG trinucleotide repeat expansion in the huntingtin (*HTT*) gene (Huntington’s Disease Collaborative Research Group, 1993). The resulting mutant HTT (mHTT) protein disrupts multiple cell signaling pathways and protein-protein interactions, leading to altered cortico-striatal (CS) transmission, synaptic loss, and degeneration of GABA-ergic striatal medium spiny neurons (MSNs; Reiner et al., 1988; Albin et al., 1992; Richfield et al., 1995; Raymond et al., 2011; Saudou and Humbert, 2016; Raymond, 2017; Tyebji and Hannan, 2017). Neuronal dysfunction and degeneration have also been reported in other brain regions including the cortex (Rüb et al., 2016). HD diagnosis usually occurs during mid-life at the time of motor symptom emergence (Roos, 2010). Despite this, studies show that cognitive, behavioral, and brain connectivity abnormalities are present decades before clinical onset in premanifest mutation carriers and that by the time of overt motor disturbances, there is significant irreversible neuronal death in the striatum (Vonsattel et al., 1985; Schippling et al., 2009; Tabrizi et al., 2009, 2011; Orth et al., 2010; Waldvogel et al., 2015). Thus, neuroprotective therapies must be administered early in the disease time course such that synaptic dysfunction can be corrected before cell death. However, strategies aimed at restoring the normal function of surviving neurons at later disease stages may still offer additional benefit.

MSNs receive high levels of excitatory glutamatergic input and trophic support (i.e., brain-derived neurotrophic factor, BDNF) from cortical afferents (McGeorge and Faull, 1989). mHTT causes both pre- and postsynaptic dysfunction at the CS synapse which contributes to the selective vulnerability of MSNs in HD (Tyebji and Hannan, 2017). MSNs express N-methyl D-aspartate receptors (NMDARs) at CS postsynaptic sites. When activated, synaptic NMDARs initiate pro-survival signaling and upregulation of genes involved in synaptic maintenance and neuroprotection (Hardingham et al., 2002; Bading, 2017). Conversely, extrasynaptic GluN2B (exGluN2B) subunit-containing NMDAR activity promotes cell death processes and antagonism of synaptic signaling (Hardingham et al., 2002; Bading, 2017). Extensive research using the YAC128 full-length, human mHTT transgenic mouse model of HD has demonstrated enhanced exGluN2B surface expression, activity, and associated excitotoxic cell death signaling before neurodegeneration or synaptic spine loss in YAC128 MSNs (Fan et al., 2007, 2009; Okamoto et al., 2009; Milnerwood et al., 2010, 2012; Gladding et al., 2012, 2014). These effects are specific to mHTT expression and not observed in transgenic human wild-type (wt)HTT-expressing YAC18 mice. GluN2B dysfunction is also dependent on the mHTT protein being cleavable at the caspase cleavage site D586, as rendering mHTT resistant to cleavage by mutating this site prevents enhanced exGluN2B surface expression and confers resistance to NMDAR excitotoxicity (Graham et al., 2006; Milnerwood et al., 2010). Preferential blockade of exNMDARs *in vivo* with low-dose memantine rescues motor deficits and neuropathology, restores striatal survival signaling, and normalizes exGluN2B levels in YAC128 mice (Okamoto et al., 2009; Milnerwood et al., 2010; Dau et al., 2014).

Increased activation of exNMDARs is a common pathological event across other major neurological disorders, including Alzheimer’s disease and ischemic stroke (Parsons and Raymond, 2014; Bading, 2017). This enhanced receptor activity causes activation of neuronal death signaling *via* p38 mitogen-activated protein kinase (MAPK), c-Jun terminal kinase (JNK), neuronal nitric oxide synthase (nNOS), caspases, and calpains, as well as shut-off of the pro-survival extracellular signal-regulated kinase (ERK) and nuclear-phosphorylated cAMP response-element binding protein (CREB) signaling, leading to synaptic dysfunction (Lai et al., 2014). Despite these compelling data, direct therapeutic targeting of NMDARs has proven to be largely ineffective in clinical trials for ischemia, likely due to partial suppression of pro-survival synaptic NMDAR activity as a result of poor compound selectivity for extrasynaptic receptor populations (Ikonomidou and Turski, 2002). For this same reason, chronic use of an NMDAR antagonist for the treatment of HD could produce adverse outcomes. These disappointing clinical trial results necessitate an alternate strategy to modulate pathological exGluN2B receptor activity while preserving synaptic function and survival signaling.

Death-associated protein kinase 1 (DAPK1) is a calcium/calmodulin-activated serine/threonine kinase that is highly expressed during late embryonic development (Deiss et al., 1995; Cohen et al., 1997; Yamamoto et al., 1999). DAPK1 was initially identified as a positive mediator of apoptosis and later as a regulator of exGluN2B function (Deiss et al., 1995; Cohen et al., 1997; Tu et al., 2010). Specifically, during ischemic excitotoxicity in the adult brain, DAPK1 becomes activated *via* S308 dephosphorylation and is recruited to exGluN2B complexes, where it phosphorylates the intracellular GluN2B C-terminal domain at S1303, increasing receptor conductance (Tu et al., 2010). S1303 phosphorylation has also been associated with enhanced GluN2B surface expression in neurons (Sanz-Clemente et al., 2013), suggesting that DAPK1 may act as a signal amplifier for exGluN2B under pathological conditions. Accordingly, numerous genetic and pharmacological DAPK1-targeting approaches have demonstrated neuroprotective and synapto-protective benefits in animal models of stroke, excitotoxicity, Alzheimer’s disease, Parkinson’s disease, and chronic stress (Li et al., 2018; Kim et al., 2019; Su et al., 2019).

In the present study, we investigate whether DAPK1 dysregulation contributes to enhanced exGluN2B function in HD mice. Furthermore, we assess the neuroprotective therapeutic efficacy of small molecule DAPK1 inhibition for the prevention of biochemical and synaptic abnormalities in YAC128 neurons.

## Materials and Methods

### Mice

YAC128 and wild-type (WT) littermate mice were maintained on the FVB/N background and experiments were performed according to protocols approved by the University of British Columbia Animal Care Committee (Protocol numbers A16-0130 and A16-0206). Heterozygous line 53 YAC128 mice or line W13 C6R mice (YAC128 mice bearing a D586A point mutation rendering mHTT resistant to caspase cleavage at this site) and their WT littermates were used for all experiments except neuronal culture transfections. For these experiments, homozygous line 55 YAC128 and FVB/N mice were used to remain consistent with previously published work (Milnerwood et al., 2012) as well as to improve culture health by avoiding an overnight genotyping step. For all cohorts, approximately equal numbers of male and female mice were used, and no differences in results were observed when assessed in each sex individually. All animals were sacrificed using CO_2_.

### Total Cell Lysis and Western Blotting

Cortical or striatal total lysates were prepared by homogenizing snap-frozen tissue in stringent lysis buffer (20 mM HEPES pH 7.4, 150 mM NaCl, 40 mM β-Glycerophosphate, 10 mM NaF, 1% Triton-X, 1% SDS, 0.5% sodium deoxycholate) with protease and phosphatase inhibitors freshly added [1X Complete Protease Inhibitor Cocktail (Roche; Cat# 11697498001), 5 μM zVAD, 1 μg/ml Pepstatin A, 1 mM sodium orthovanadate]. Protein samples were sonicated, centrifuged to remove cellular debris, and quantified by DC assay (BioRad; Cat# 5000112). Equal amounts of protein from each sample were run by SDS-PAGE on 8 or 10% acrylamide gels and transferred to 0.45 μm nitrocellulose membranes. Membranes were blocked in 5% skim milk powder in TBST (0.1% Tween-20) for 1 h at room temperature (RT), blotted in primary antibody in 3% BSA/TBST for 2 h at RT, washed 4× in TBST, incubated with fluorescent secondary antibody for 1 h at RT, washed, and scanned with an LI-COR Odyssey imaging system. Band intensities were quantified in Image Studio Lite using top-bottom background subtraction. Measurements for proteins of interest were normalized to the intensity of a loading control which was probed on the same blot. All Western blot images within each figure panel were run on the same gel and cropped from a single scan image at identical brightness/contrast adjustments. Molecular weights (in kDa) are noted on the right side of each cropped blot image.

### Subcellular Fractionation

Subcellular fractionations from brain tissue were performed as previously described to isolate synaptic (post-synaptic density, PSD) and extrasynaptic (non-PSD) membranes, as well as nuclear fractions (Milnerwood et al., 2010). Striatal non-PSD preparations consistently produced low protein yield and unreliable detection of GluN2B phosphorylation which was not consistent between technical replicates from the same biological starting sample. Therefore, we chose an alternative method for evaluation of striatal “non-synaptic” pS1303 levels which has previously been described (Gladding et al., 2014), and provided much more reliable results due to decreased protocol time and lesser centrifugation and wash steps (and thus reduced protein/volume loss). We used the term “non-synaptic” throughout the “Results and Discussion” sections to distinguish where this modified method was used in place of standard fractionation (in which case the term “extrasynaptic fraction” is used). To generate non-synaptic fractions, tissue samples were homogenized in a gentle lysis buffer (20 mM HEPES pH 7.4, 150 mM NaCl, 40 mM β-Glycerophosphate, 10 mM NaF, 1% Triton-X) containing fresh protease and phosphatase inhibitors, but no SDS or sodium deoxycholate. This buffer is unable to lyse Triton-insoluble PSD membranes. Thus, after homogenization and high-speed centrifugation, the remaining supernatant was assumed to contain cytosol and non-synaptic membranes, including endosomes, while the insoluble pellet contained the synaptic PSD. Since GluN2B is a transmembrane protein, any GluN2B in the final non-synaptic protein sample was assumed to be present in membranes, rather than the cytosol. However, these non-synaptic membranes could represent either plasma membrane, internalized membrane vesicles, or both. To validate both our standard subcellular fractionation protocol as well as our modified lysis protocol, we confirmed that both methods resulted in the enrichment of the synaptic proteins PSD95 and GluN2B in the PSD fractions, as expected (Supplementary Figure 1). Synaptic, extrasynaptic, and “non-synaptic” protein fractions were quantified and used for Western blotting as described above. Antibodies and conditions used for Western blotting are listed in the following table:

**Table:** 

Target	Host	Company	Catalog#	Dilution
β-actin	Rabbit	Cell Signaling	4967	1:2,000
CaMKIIα	Mouse	Cell Signaling	50049	1:2,000
CaMKII-pT286	Rabbit	R&D Systems	PPS002	1:2,000
DAPK1	Rabbit	Sigma–Aldrich	D1319	1:2,000
DAPK1	Mouse	Invitrogen	MA1–24696	1:250
DAPK1-pS308	Mouse	Sigma–Aldrich	D4941	1:400
GluN2B	Mouse	Invitrogen	MA1–2014	1:1,000
GluN2B-pS1303	Rabbit	Millipore	07–398	1:1,000

### Co-immunoprecipitation

Cortical tissue was homogenized in a gentle lysis buffer (50 mM Tris, 150 mM NaCl, 1% Igepal) with fresh protease inhibitors and pre-cleared with 10 μl of Protein G Dynabeads (Invitrogen; Cat# 10004D) for 1.5 h at 4°C. During the pre-clear step, 15 μl of Dynabeads per sample were washed once in lysis buffer, blocked with 3% BSA in lysis buffer, and conjugated to primary rabbit anti-DAPK1 antibody or control rabbit IgG (2.5 μg per sample) in blocking solution for 1 h at RT. Beads were washed twice in lysis buffer, added to pre-cleared lysates, and rotated overnight at 4°C. Beads were washed three times for 10 min in lysis buffer and protein was eluted in 1× NuPAGE LDS sample buffer (Invitrogen; Cat# NP0007) with 100 mM DTT at 70°C for 10 min. Samples were run by SDS–PAGE alongside previously set-aside input controls, transferred to nitrocellulose membranes, and blotted for DAPK1 and GluN2B using mouse primary antibodies. Co-immunoprecipitated GluN2B band intensity was normalized to the amount of immunoprecipitated DAPK1 for each sample.

### *In vivo* Memantine Treatments

Male WT and female YAC128 (line 53) mice were set up in mating pairs and provided either regular drinking water or water containing memantine. The concentration of memantine was calculated based on the weight of the female mouse and the estimated average consumption of 5 ml per day to administer a dose of 2 mg/kg/day. Final memantine concentrations were approximately 12 mg/l. Females were separated from males after 1 week, and pregnant females remained on either water or memantine treatment until pups were weaned at P20. At this time, male and female pups were separated and the average pup weight per cage was used to calculate a 1 mg/kg/day dose, assuming an average consumption of 4 ml per day per pup. Final memantine concentrations for pup treatment were approximately 2.5 mg/l. At 1 month of age, mice were sacrificed and brain tissue was collected for subcellular fractionation and Western blotting.

### Neuronal Culture Setup and Transfections

Autoclaved 12 mm No. 1 glass coverslips (Marienfeld Superior) were transferred to 24-well plates, and coated with 100 μg/ml high molecular weight poly-D-lysine (PDL) hydrobromide (Sigma–Aldrich; Cat# P6407) at RT overnight, washed 4x thoroughly with water, and air-dried before use. E16.5–17.5 embryos were removed from pregnant female mice, and brains were kept overnight at 4°C in Hibernate-E supplemented with L-glutamine (0.5 mM, Gibco; Cat# 25030) and B27 (Gibco; Cat# 17504) while excess embryonic tissue was used to perform genotyping. For dendritic spine experiments, cortical and striatal tissues were dissected separately in ice-cold Hank’s Balanced Salt Solution (Gibco; Cat# 14170). Dissected tissue was dissociated by gently pipetting once with a P1000 pipette, centrifuged, and enzymatically digested in 0.05% trypsin-EDTA (Gibco; Cat# 25300) for 8 min at 37°C. Trypsin was inactivated with 10% FBS in neurobasal medium (NBM; Gibco; Cat# 21103). Cells were then triturated gently 3–5× incomplete NBM (supplemented with L-glutamine, penicillin/streptomycin, and B27) containing DNase 1 (0.08 mg/ml) for further dissociation. Cells were centrifuged, resuspended in complete NBM, counted, and plated at a 1:3 cortical:striatal ratio with a final density of 160,000 cells per well. For evaluation of exogenous YFP-GluN2B surface expression, striatal neurons were nucleofected with a YFP-GluN2B construct (2 μg; a gift from Dr. Ann Marie Craig, University of British Columbia, Vancouver, BC, Canada) before being mixed with cortical neurons at a 1:1 ratio and plated in 500 μl 10% FBS/DMEM. The media was replaced with 500 μl of warm complete NBM after 3–4 h and topped up to 1 ml the following day. For biochemical experiments, cortical and striatal tissue were kept together during the culture process and seeded in 6-well PDL-coated plates at a density of 1 million cells per well. We estimate that the cortical:striatal ratio in these cultures was approximately 3:1–4:1. All cultures were fed with fresh complete NBM (25% well volume) every 6–7 days until DIV14 and every 3–4 days afterward unless undergoing drug treatments.

### Neuronal Culture Drug Treatments and Lysis

For imaging experiments, neurons grown on coverslips were treated every day from either DIV14 or DIV18 until DIV20. Ifenprodil hemitartrate (Tocris Bioscience; Cat# 0545) and memantine hydrochloride (Tocris Bioscience; Cat# 0773) were dissolved in sterile H_2_O and used at a final treatment concentration of 3 μM. The DAPK1 inhibitor TC-DAPK6 (DKI; Tocris Bioscience; Cat# 4301) was dissolved in DMSO and used at a final concentration of 1 μM (0.01% DMSO). Cultures were fixed and immunostained at DIV21.

For biochemistry, culture media was replaced with conditioned media containing 10 μM DKI or DMSO (0.1%) for 1 h. Cells were collected by scraping immediately following treatment, centrifuged, and frozen at −20°C for later processing. Pellets were lysed by pipetting in gentle lysis buffer to solubilize only non-synaptic proteins. Samples underwent SDS–PAGE and Western blotting as described above for the detection of protein and phosphorylation levels.

### Immunocytochemistry, Microscopy, and Image Analysis

For spine experiments, neurons on coverslips were fixed at DIV21 for 15 min at RT in 4% paraformaldehyde/PBS. Coverslips were washed 3× in PBS after fixation and between all subsequent incubation steps. Samples were incubated at −20°C in methanol for 5 min, permeabilized in 0.03% Triton-X/PBS at RT for 5 min, blocked in 0.2% gelatin/PBS at RT for 30 min, incubated with primary antibody against DARPP32 in blocking solution overnight at 4°C and fluorescent secondary antibody for 1.5 h at RT. Coverslips were mounted on microscope slides using Prolong Gold Antifade Reagent with DAPI (Invitrogen; Cat# P36935). Z-stack images of step size 0.6 μm were acquired using a Leica SP8 confocal microscope with a 63× objective lens. Representative MSNs for imaging were selected based on a healthy nuclear appearance and high DARPP32 expression level. In ImageJ, files were converted to 2D images with the maximum intensity Z-projection option, background-subtracted with a rolling ball radius of 35 pixels, and smoothed using the de-speckle function. De-speckling was necessary, as it dramatically improved the efficiency and accuracy of automatic spine detection in NeuronStudio (Version 0.9.92). Images were imported into NeuronStudio for semi-automatic spine identification and classification using at least three different secondary or tertiary dendritic segments per neuron.

Exogenous YFP-GluN2B surface expression was assessed as previously described (Milnerwood et al., 2012). In transfected, unstained cultures, the basal fluorescence of the YFP (which is N-terminal and thus expressed on the extracellular domain of GluN2B) is extremely low and difficult to detect. Transfected cultures grown on coverslips were thus live-stained at DIV21 to detect surface GluN2B by incubating with primary chicken GFP antibody in conditioned media for 10 min in a 37°C CO_2_ incubator. Cells were rinsed once with warm conditioned media, fixed, and incubated with Alexa Fluor 488-conjugated goat anti-chicken secondary antibody for 1.5 h at RT. Samples were subsequently permeabilized, blocked, and stained as described above for internal YFP and VGLUT1 using rabbit and guinea pig primary antibodies, respectively. This was followed by fluorescent secondary antibody staining (Alexa Fluor 568-conjugated goat anti-rabbit and Alexa Fluor 647-conjugated goat anti-guinea pig). In this way, surface YFP-GluN2B could be measured *via* the Alexa Fluor 488 (green) intensity, while internal YFP-GluN2B could be detected by measuring the intensity of Alexa Fluor 568 (red). Coverslips were mounted on slides and transfected cells were imaged at 63× objective magnification in three channels at constant laser intensity across samples. Three representative secondary or tertiary dendritic segments were selected and the mean intensity value from each GFP channel within each selection was measured and averaged to generate a surface-to-internal ratio value as a measure of surface expression. The numbers of total YFP-GluN2B punctae and punctae colocalized with VGLUT1 were counted manually and normalized to the dendritic area to yield GluN2B punctae and synapse densities. For all imaging experiments, a total of at least 24 neurons from three individual culture batches were imaged. Primary antibodies and dilutions used for immunocytochemistry experiments are listed in the table below. Alexa Fluor goat secondary antibodies directed to the appropriate primary antibodies were used at a 1:500 dilution. All imaging and analyses were performed with the researcher blinded to experimental conditions.

**Table:** 

Target	Host	Company	Catalog#	Dilution
DARPP32	Rat	R&D Systems	MAB4230	1:500
GFP	Chicken	Abcam	Ab13970	1:1,000
GFP	Rabbit	Synaptic Systems	132 002	1:500
VGLUT1	Guinea Pig	Millipore	AB5905	1:4,000

### COS-7 Cell Culture, Transfection, and Immunocytochemistry

COS-7 cells were grown at 37°C and 5% CO_2_ in high glucose DMEM (Gibco; Cat# 11965) supplemented with 10% FBS (Gibco; Cat# 26140). Cells were transiently co-transfected with constructs encoding GluN1a (A gift from Dr. Lynn Raymond, University of British Columbia), GFP-tagged GluN2B (a gift from Dr. Katherine Roche, National Institutes of Health; Sanz-Clemente et al., 2013), and C-terminal FLAG-tagged DAPK1 (a gift from Dr. Ruey-Hwa Chen, Academia Sinica; You et al., 2017) at a 2:2:1 ratio using X-tremeGENE 9 transfection reagent (Roche; Cat# 6365809001). For relevant experiments, DMSO or DKI (10 μM) were added to cell culture media at the time of transfection. Live GFP-GluN2B surface staining was performed 24 h post-transfection, followed by immunocytochemistry for internal GFP-GluN2B and DAPK1-FLAG, as described in the previous section. Microscopy and image analysis were performed as done for neuronal culture experiments, except surface expression was quantified within three representative surfaces/cytosolic non-nuclear regions within each imaged cell identified as positively co-expressing surface GFP-GluN2B and DAPK1-FLAG. Primary antibodies and dilutions used for immunocytochemistry experiments are listed in the table below. Alexa Fluor goat secondary antibodies directed to the appropriate primary antibodies were used at a 1:500 dilution (488-conjugated goat anti-chicken, 568-conjugated goat anti-mouse, 647-conjugated goat anti-rabbit).

### Azidohomoalanine (AHA) Pulse-Chase Assay

Pulse-chase assays were performed using bio-orthogonal labeling with the methionine analog azido-homoalanine (AHA), as previously described (Dieterich et al., 2007; Ullrich et al., 2014; Wang et al., 2017). COS-7 cells were grown at 37°C in DMEM supplemented with 10% FBS. Cells were transiently co-transfected with GluN1a, GFP-tagged GluN2B, and FLAG-tagged DAPK1 DNA constructs as described above. 24 h post-transfection, cells were deprived of methionine and cysteine and incubated with 50 μM AHA and non-labeled cysteine for 1 h (pulse). After the pulse, cells were washed with PBS and incubated with regular media (chase). Cells were harvested after 0, 0.5, 1, 2, 3, 5, 8, 10, or 24 h of chase time and lysed in RIPA buffer containing protease and phosphatase inhibitors. GFP-tagged GluN2B and FLAG-tagged DAPK1 were immunoprecipitated from cell lysates by overnight incubation with Protein G Dynabeads and goat anti-GFP polyclonal (Eusera; Cat# EU4) or mouse anti-FLAG monoclonal (Millipore Sigma–Aldrich; Cat# F1804) antibodies, respectively. Bio-orthogonal click chemistry of AHA-labeled proteins with biotin-PEG4-alkyne (Sigma–Aldrich; Cat# 764213) was subsequently performed. GluN2B and DAPK1 immunoprecipitates were adjusted to 1% SDS and incubated with 100 mM Tris (benzyl-triazolyl methyl)amine (TBTA), 1 mM CuSO_4_, 1 mM Tris-carboxyethyl phosphine (TCEP), and 100 mM azido-biotin at 37°C in darkness for 30 min. Samples were washed and boiled to remove protein from the beads. Total GluN2B and DAPK1 were detected by Western Blot analysis with mouse anti-GluN2B (Thermo Fisher Scientific; Cat# MA1–2014; 1:800) or rabbit anti-DAPK1 (Millipore Sigma; Cat# D1319; 1:1,000), respectively. GluN2B or DAPK1 bio-orthogonally labeled with biotin was detected with Alexa Fluor 680 conjugated to streptavidin (Life Technologies; Cat# S-32358). Protein turnover was determined by calculating the ratio of biotin-labeled protein to total protein signal and normalizing to *t* = 0 h.

### RNA Extraction, cDNA Synthesis, and qPCR

To assess gene expression, a small piece of anterior cortical tissue was cut and RNA was extracted using an RNeasy Mini Kit (Qiagen; Cat# 74106) and RNase-free DNase set (Cat# 79254). cDNA was synthesized with the Superscript III First-Strand system according to manufacturer instructions (Life Technologies; Cat# 18080–051). Quantitative RT-PCR reactions were prepared in MicroAmp Fast optical 96-well reaction plates (Applied Biosystems; Cat# 4346907) with Power SYBR™ Green PCR Master Mix (Thermo Fisher Scientific; Cat# 4368706), and run on the Applied Biosystems 7500 Fast Real-Time system. *Dapk1* expression was quantified based on ΔΔCt values using the housekeeping gene *Rpl13a. Dapk1* primer sequences: 5′- GTCCGCTACCTCTGTTTGATG-3′ and 5′-GTGTCCTTCCGCAGTCTTG-3′. *Rpl13a* primer sequences: 5′-GGAGGAGAAACGGAAGGAAAAG-3′ and 5′-CCGTAACCTCAAGATCTGCTTCTT-3′.

### Intranasal Administration of DAPK1 Inhibitor TC-DAPK6 (DKI)

Mice (4 weeks old) were anesthetized with ketamine and xylazine (100/10 mg/kg i.p.) and then placed in a supine position with their noses upright and heads flat on the bench surface. The DAPK1 inhibitor TC-DAPK6 (DKI) was dissolved in 50% PEG, 10% DMSO, 40% NaCl (0.9% w/v), and used at a final concentration of 1.67 μM. A 10 μl Hamilton syringe with a blunted needle tip was used to administer either vehicle or DKI in 2 μl increments per nare every 4 min, alternating sides until the total dose (30 μl; 50 nmol) was given. Mice remained supine for 30 min after intranasal administration. After 6 h, mice were sacrificed and samples were collected for subcellular fractionation experiments.

### Statistical Analysis

GraphPad Prism 5 was used for all statistical analysis and graph preparation. Data are presented as mean ± SEM. Two-tailed Student’s *t*-test, one-way ANOVA, or two-way ANOVA statistical tests with *post hoc* analysis (Bonferroni or Dunnett) were used for all experiments with an alpha level set to 0.05. In some cases, two-tailed *t*-tests were performed between specific groups after an ANOVA test when a planned comparison was established during the experimental design process. Significance for these tests is noted with the “#” symbol instead of “*”. For all experiments, data were tested to ensure normality and equal variance between groups. Figures were generated in Adobe Photoshop CS5.

## Results

### DAPK1 Expression and Activation Are Dysregulated in Affected Regions of the YAC128 HD Mouse Brain

**Figure 1:**
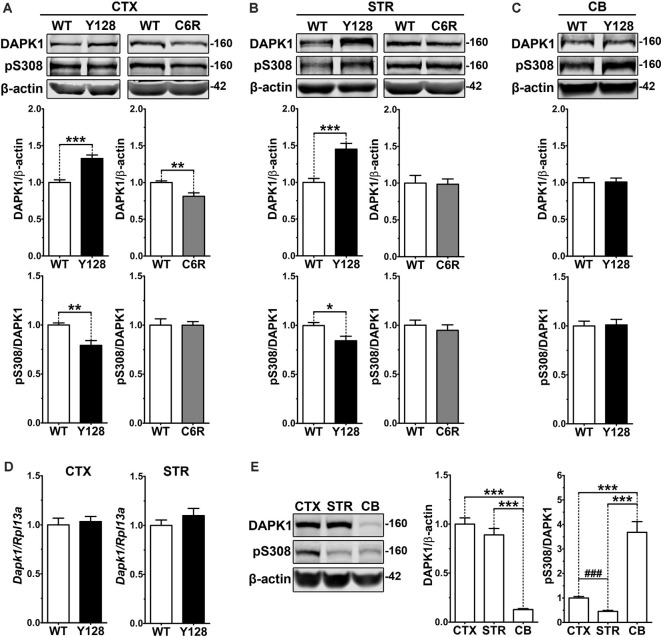
Increased DAPK1 protein expression and activation occur in affected regions of the YAC128 brain and require mHTT cleavage at D586. **(A)** Cortical (CTX), **(B)** striatal (STR), or **(C)** cerebellar (CB) tissues from 1-month-old WT, YAC128 (Y128; line 53), and C6R (line W13) mice were lysed in a stringent buffer and total lysate was assessed by Western blotting for DAPK1, pS308, and β-actin protein levels. Data are normalized to WT (*n* = 6–12 biological replicates, two technical replicates each; Student’s *t*-test, *p < 0.05, **p < 0.01, ***p < 0.001). **(D)** *Dapk1* mRNA expression in WT and YAC128 brains at 1 month of age was quantified by qPCR. Data are normalized to WT (*n* = 6 biological replicates; Student’s *t*-test). **(E)** Cortical, striatal, and cerebellar tissues from 1-month-old WT FVB mice were evaluated by Western blot for DAPK1, pS308, and β-actin protein levels. Data are normalized to CTX values (*n* = 8 biological replicates, two technical replicates each; Student’s *t*-test, ^###^*p* < 0.001; one-way ANOVA with Bonferroni *post hoc* analysis, ***p < 0.001).

Given the established role of exNMDAR excitotoxicity in HD, we sought to evaluate whether DAPK1 is dysregulated in YAC128 mice. One-month-old animals were used because YAC128 mice at this age demonstrate enhanced sensitivity to excitotoxins and elevated striatal exGluN2B, before behavioral or neuropathological phenotypes (Graham et al., 2009; Milnerwood et al., 2010). We observed significantly increased DAPK1 protein levels in total lysates from both the cortex and striatum of YAC128 mice, as well as reduced site-specific phosphorylation at the autoinhibitory S308 residue (normalized to protein level), indicating greater kinase activation (Figures 1A,B). These changes were not observed in the cerebellum, which is largely spared in HD (Vonsattel et al., 2008; Figure 1C). We also did not observe any differences in DAPK1 protein level or phosphorylation in the cortex of transgenic human wtHTT-expressing YAC18 control mice (Supplementary Figure 2), signifying that these changes are not due to general overexpression of human HTT. When we evaluated *Dapk1* mRNA levels, we found no differences between genotypes (Figure 1D), suggesting altered DAPK1 protein stability or turnover in YAC128 brains, as opposed to elevated transcription. Interestingly, in 1-month-old WT FVB animals, DAPK1 expression was significantly higher in cortical and striatal tissues when compared directly to the cerebellum (Figure 1E). Furthermore, phosphorylation of DAPK1 at S308 was reduced in the striatum compared to the cortex and was dramatically increased in the cerebellum (Figure 1E). Thus, in the unaffected brain, DAPK1 expression and activation are highest in the regions most affected by HD.

The C6R mouse model expresses the YAC128 transgene bearing point mutations at residues D586 and D589, rendering mHTT resistant to caspase cleavage at D586 (Graham et al., 2006). Unlike YAC128 mice, C6R mice are resistant to excitotoxicity, do not demonstrate enhanced exNMDAR activity, and display no motor deficits or neuropathology, indicating that mHTT cleavage is a critical contributor to exNMDAR dysfunction in HD mice (Graham et al., 2006; Milnerwood et al., 2010). We found that DAPK1 expression is reduced in the cortex and unaltered in the striatum of 1-month-old C6R mice, and pS308 levels are comparable to WT in both brain regions (Figures 1A,B), suggesting that D586 cleavage is required for mHTT-induced dysregulation of DAPK1.

### Phosphorylation of exGluN2B at the DAPK1 Site Is Increased in the YAC128 Brain

**Figure 2:**
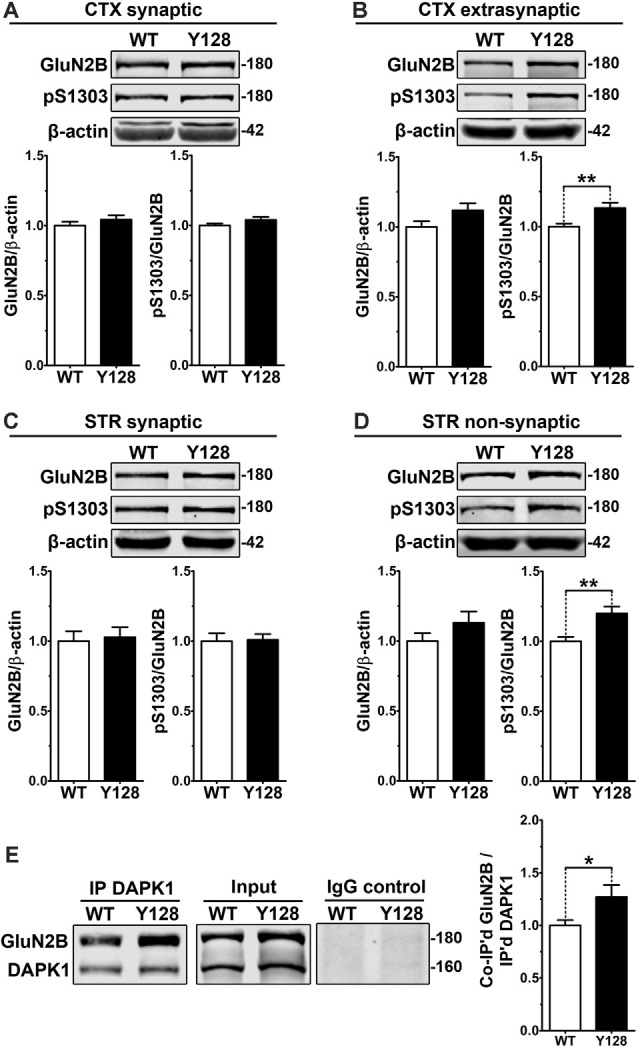
Extrasynaptic GluN2B S1303 phosphorylation and interaction with DAPK1 are elevated in the YAC128 brain. Cortical or striatal tissues were subjected to subcellular fractionation to yield **(A,C)** synaptic (PSD), **(B)** cortical extrasynaptic (non-PSD), or **(D)** striatal “non-synaptic” membrane fractions, which were run by SDS–PAGE and Western blotting for GluN2B expression and S1303 phosphorylation (*n* = 6–14 biological replicates, two technical replicates each; Student’s *t*-test, *p < 0.05, **p < 0.01). **(E)** DAPK1 was immunoprecipitated from WT and YAC128 cortical lysates, followed by the detection of co-immunoprecipitated GluN2B by Western blot. All sample blot images in panel **(E)** are cropped from the same Western blot membrane which contained multiple biological replicates run side-by-side. Data are normalized to WT values (*n* = 14 biological replicates, Student’s *t*-test, *p < 0.05, **p < 0.01).

DAPK1 interacts with and phosphorylates exGluN2B at S1303, which contributes to the amplification of receptor function and surface expression (Tu et al., 2010; Sanz-Clemente et al., 2013). We hypothesized that an increase in DAPK1 expression and activity in the YAC128 brain would correlate with enhanced phosphorylation at this GluN2B residue. We evaluated GluN2B and pS1303 levels in synaptic (post-synaptic density, PSD) and extrasynaptic (non-PSD) membrane compartments from 1-month-old WT and YAC128 tissues. We observed an increase in pS1303 in YAC128 cortices, which was specific to the extrasynaptic compartment, and not observed in synaptic fractions (Figures 2A,B). We did not detect altered GluN2B or pS1303 levels in YAC128 striatal synaptic fractions (Figure 2C). However, using a previously-described modified method to evaluate striatal “non-synaptic” protein (Gladding et al., 2014), we observed a significant increase in non-synaptic pS1303 in the YAC128 striatum (Figure 2D).

DAPK1 has been shown to interact more strongly with GluN2B receptors *in vivo* under pathological conditions and when S308 is dephosphorylated (Tu et al., 2010; Goodell et al., 2017). Furthermore, phosphorylation of GluN2B at S1303 enhances the DAPK1-GluN2B interaction (Goodell et al., 2017). We hypothesized that a chronic elevation in DAPK1 expression, activation, and exGluN2B pS1303 levels would be associated with an enhanced basal DAPK1-GluN2B interaction in YAC128 brains. In non-synaptic compartments, which were isolated using a gentle lysis buffer, we observed an increased interaction between GluN2B and DAPK1 in the YAC128 cortex (Figure 2E).

Even though GluN2B pS1303 was elevated specifically in the extrasynaptic compartment in YAC128 brains, we found that DAPK1 expression itself was increased in both synaptic and extrasynaptic membrane fractions from the YAC128 cortex and striatum (Supplementary Figure 3). Thus, although additional consequences of increased DAPK1 at the synapse may exist, the effect on GluN2B phosphorylation at S1303 appears specific to the extrasynaptic receptor population.

Calcium/calmodulin-dependent protein kinase II (CaMKII) is a DAPK1-related kinase with established roles in synaptic signaling, neuronal plasticity, and receptor regulation (Shonesy et al., 2014). Similar to DAPK1, CaMKII has been implicated in ischemic excitotoxicity related to NMDAR activation, and also phosphorylates GluN2B at S1303 (Omkumar et al., 1996; Coultrap et al., 2011), influencing receptor surface expression (Sanz-Clemente et al., 2013). However, we found that CaMKII expression and autonomous activation measured by phosphorylation at T286 were unaltered in total, synaptic and extrasynaptic compartments of the YAC128 cortex and striatum (Supplementary Figure 4).

### *In vivo* exNMDAR Blockade Normalizes DAPK1 Expression, Activation, and exGluN2B pS1303 in the YAC128 Cortex

**Figure 3:**
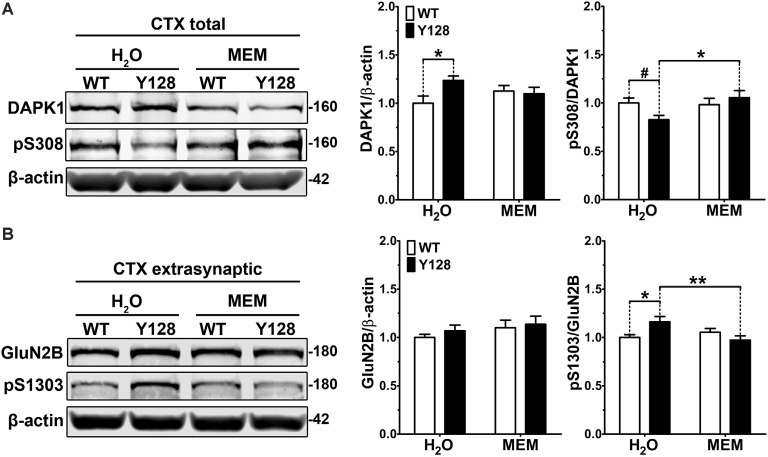
Low-dose memantine normalizes cortical DAPK1 activation and extrasynaptic pS1303 levels in YAC128 mice. Cortical tissues from 1-month-old WT and YAC128 mice treated with low-dose memantine from conception were processed to obtain **(A)** total or **(B)** extrasynaptic (non-PSD) membrane protein fractions. Samples were processed for DAPK1 or GluN2B protein expression and phosphorylation levels by Western blot. Data are normalized to WT H_2_O values (*n* = 8 biological replicates, two technical replicates each; two-way ANOVA with Bonferroni *post hoc* analysis, *p < 0.05, **p < 0.01; Student’s *t*-test, ^#^*p* < 0.05; two-way ANOVA interaction *p < 0.05 for DAPK1 protein level and pS308, two-way ANOVA interaction **p < 0.01 for pS1303).

It was previously shown that the treatment of cortical neurons with NMDA caused activation of DAPK1, phosphorylation of GluN2B at S1303, and increased exNMDAR-mediated calcium transients (Tu et al., 2010; Fan et al., 2014). These effects were abolished by the NMDAR antagonist AP5 and by genetic depletion of DAPK1, indicating that DAPK1 is activated downstream of NMDARs and subsequently augments exGluN2B phosphorylation and conductance (Tu et al., 2010). In YAC128 mice, low-dose treatment with memantine, which preferentially blocks exNMDAR channels, normalizes striatal cell survival (pCREB) and death (p-p38 MAPK) signaling, returns exGluN2B expression to WT levels, and prevents motor function decline and neuropathology (Okamoto et al., 2009; Milnerwood et al., 2010; Dau et al., 2014). We thus hypothesized that a positive feedback loop may be pathologically amplified in HD mice, whereby elevated DAPK1 activation in YAC128 brains may both result from and contribute to increased exNMDAR function. To address the former, we treated WT and YAC128 mice with low-dose memantine from conception by administering memantine in the drinking water to breeders and subsequently to pups once weaned. Tissues were collected for biochemistry at 1 month of age. We found that memantine treatment normalized DAPK1 S308 phosphorylation in YAC128 total cortical lysate to WT levels (Figure 3A). Treatment also normalized elevated YAC128 exGluN2B S1303 phosphorylation (Figure 3B). These findings indicate that DAPK1 dephosphorylation and enhanced pS1303 levels may occur downstream of exNMDAR activity in the YAC128 cortex.

### DAPK1 Promotes mHTT-induced exGluN2B Phosphorylation and Surface Expression

**Figure 4:**
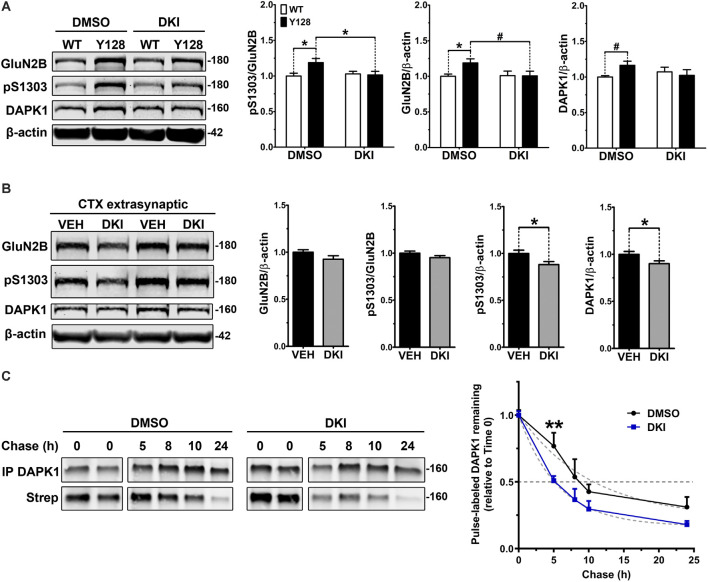
DAPK1 inhibition normalizes extrasynaptic GluN2B phosphorylation and DAPK1 protein levels in the YAC128 model. **(A)** Corticostriatal primary cultures from WT and YAC128 embryos were treated at DIV21 with DMSO (0.1%) or a DAPK1 inhibitor (DKI, 10 μM) for 1 h followed by lysis in a gentle buffer to solubilize non-synaptic protein. Lysates were processed by Western blot to detect GluN2B, pS1303, and DAPK1 levels. Data are normalized to WT DMSO values (*n* = 8 biological replicates; two-way ANOVA with Bonferroni *post hoc* analysis, *p < 0.05; Student’s *t*-test, ^#^*p* < 0.05). **(B)** Four-week-old YAC128 mice were treated intranasally with 50 nmol of DAPK1 inhibitor (DKI; TC-DAPK6) or vehicle control (VEH). After 6 h, cortical extrasynaptic fractions were isolated to measure GluN2B, pS1303, and DAPK1 levels (*n* = 9 biological replicates, two technical replicates each; Student’s *t*-test, *p < 0.05). **(C)** Quantitative measurement of DAPK1-FLAG protein turnover using bio-orthogonal labeling, FLAG immunoprecipitation, CLICK chemistry, and detection of AHA/Streptavidin (Strep)-labeled DAPK1 in transiently transfected COS-7 cells treated with DMSO or 10 μM DKI (TC-DAPK6) for 24 h (*n* = 3 for 8 h and 10 h chase time points, *n* = 7 for all other chase time points; two-way ANOVA **p < 0.01 for DKI treatment effect; Sidak’s multiple comparisons test, **p < 0.01).

Our next experiments aimed to address whether DAPK1 contributes to increased exGluN2B phosphorylation or surface expression in YAC128 neurons. Consistent with the role of DAPK1 in amplifying exGluN2B function in ischemia, genetic manipulations eliminating DAPK1 expression or kinase activity promote neuroprotection and synaptic preservation in models of stroke, excitotoxicity, and Alzheimer disease. Furthermore, a selective, small-molecule DAPK1 inhibitor (TC-DAPK6, referred to here as “DKI”) protects against glutamate toxicity in SH-SY5Y cells, enhances the maturation of primary cultured neurons, and prevents chronic stress-induced GluN2B S1303 phosphorylation, CREB dephosphorylation, and loss of brain-derived neurotrophic factor (BDNF) levels (Kim et al., 2014; Tian et al., 2014; Li et al., 2018). We used this same compound to evaluate the effect of targeting DAPK1 kinase activity on GluN2B receptors in YAC128 neurons. Primary YAC128 corticostriatal cultures exhibited increased endogenous pS1303, GluN2B, and DAPK1 levels at DIV21 compared to WT (Figure 4A). These experiments were performed using a gentle lysis buffer to only solubilize non-synaptic proteins. Treatment with DKI normalized all of these measures to WT levels (Figure 4A). We validated this finding *in vivo* by delivering DKI *via* intranasal administration to YAC128 mice at 4 weeks of age. Mice were sacrificed 6 h post-treatment and examination of extrasynaptic cortical membrane fractions indicated a trend to a reduction in GluN2B levels (Figure 4B). There was also a slight trend to decreased phosphorylation of exGluN2B at S1303 when normalized to GluN2B protein levels. Interestingly, when normalized to the β-actin loading control, pS1303 levels were significantly reduced with DKI treatment (Figure 4B). Furthermore, DKI treatment significantly reduced extrasynaptic DAPK1 protein levels (Figure 4B), thus confirming *in vivo* what we observed in DKI-treated YAC128 primary neuronal cultures. Together, this suggests that that DAPK1 activity contributes to pathologically elevated non-synaptic GluN2B and pS1303 in the presence of mHTT.

We further explored our observation that DKI normalized DAPK1 protein levels in YAC128 cultures compared to WT by assessing whether this effect was due to a treatment-induced alteration in the stability of the DAPK1 protein. We performed pulse-chase experiments with the traceable amino acid azidohomoalanine (AHA) in COS-7 cells triple-transfected with DAPK1, GluN1, and GluN2B constructs and found that DKI significantly increased DAPK1 protein turnover compared to DMSO control conditions (Figure 4C), thus supporting our hypothesis that activity of DAPK1 promotes stability of the protein.

**Figure 5:**
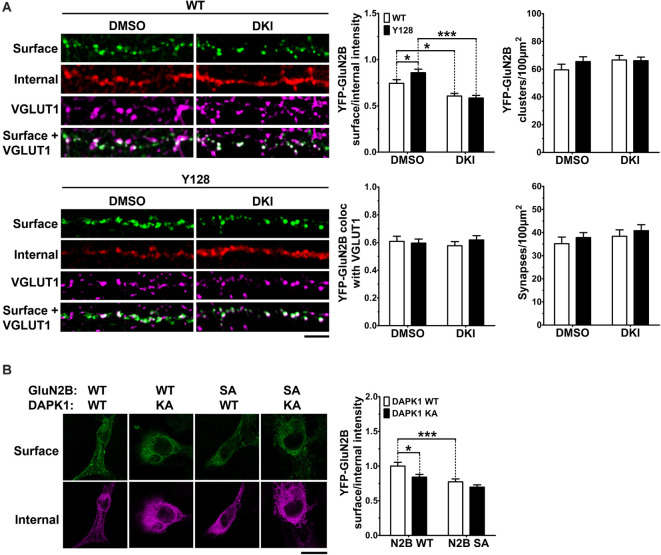
DAPK1 kinase activity promotes GluN2B surface expression *via* pS1303. **(A)** Surface expression (surface/internal YFP fluorescence ratio) and cluster analysis of transfected YFP-GluN2B was performed in 1:1 cortico-striatal co-cultured YAC128 medium spiny neurons (MSNs) treated with DMSO or the DAPK1 inhibitor TC-DAPK6 (DKI; 1 μM for 3 days, 0.01% final DMSO concentration). Absolute values were used for punctae analysis (*n* = 24–38 cells from three independent cultures; two-way ANOVA with Bonferroni *post hoc* analysis, *p < 0.05, ***p < 0.001). Scale bar = 5 μm. **(B)** Surface expression of GFP-GluN2B [WT or an S1303A (SA) mutant] in transfected COS-7 cells co-expressing GluN1 and either WT-DAPK1 or a kinase-dead DAPK1 mutant (K42A; KA; *n* = 23–30 cells from three independent culture passages; two-way ANOVA with Bonferroni *post hoc* analysis, *p < 0.05, ***p < 0.001). Scale bar = 15 μm.

Previously, an increase in surface expression (relative to internal expression) of transfected YFP-GluN2B was observed in YAC128 MSNs co-cultured with cortical neurons at a 1:1 ratio, compared to WT (Milnerwood et al., 2012). As the basal fluorescence of YFP conjugated to GluN2B is extremely low, this experiment requires amplification of the YFP by immunocytochemistry using primary antibodies and fluorophore-conjugated secondary antibodies. We confirmed the phenotype reported in Milnerwood et al., 2012 in the present study and found that inhibition of DAPK1 reduced YFP-GluN2B surface expression in MSNs of both genotypes without affecting the number of receptor clusters, percent punctae colocalization with the presynaptic marker vesicular glutamate transporter 1 (VGLUT1), or the number of synapses (YFP-GluN2B/VGLUT1 colocalized points; Figure 5A). To assess whether the effect of blocking DAPK1 activity on surface expression may be mediated *via* altered S1303 phosphorylation, we performed GluN2B surface expression analysis on COS-7 cells triple-transfected with DAPK1, GluN1, and GluN2B constructs. We found that co-expression of a kinase-dead DAPK1 mutant (K42A; KA) resulted in reduced surface expression of GluN2B compared to co-expression with WT DAPK1 (Figure 5B). As described previously (Sanz-Clemente et al., 2013), rendering S1303 phospho-deficient by mutating to alanine (S1303A; SA), also significantly decreased GluN2B surface expression (Figure 5B). Notably, the S1303A mutation occluded the effect of the DAPK1 K42A mutation. Altogether, these results demonstrate that active DAPK1-induced phosphorylation of S1303 increases exGluN2B phosphorylation and surface expression, and suggest that pathological amplification of this process may contribute to exGluN2B dysfunction in the presence of mHTT.

### DAPK1 Inhibition Prevents Dendritic Spine Instability in YAC128 MSNs

**Figure 6:**
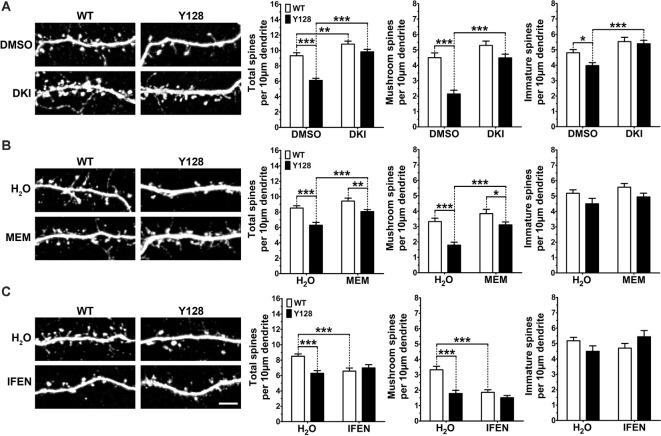
DAPK1 inhibition or extrasynaptic NMDAR blockade prevents spine instability in YAC128 MSNs. **(A)** WT and YAC128 1:3 CS co-cultures were treated from DIV14 to 21 with DMSO (0.01%) or the DAPK1 inhibitor TC-DAPK6 (DKI, 1 μM), and spine analysis was performed on DARPP32+ MSNs. **(B)** 1:3 co-cultures were treated with either water, memantine (MEM, 3 μM) or **(C)** ifenprodil (IFEN, 3 μM) from DIV14 to 21 and processed in the same way as for DKI (*n* = 24 cells from three independent cultures; Two-way ANOVA with Bonferroni *post hoc* analysis, *p < 0.05, **p < 0.01, ***p < 0.001). Scale bar = 5 μm.

MSN spine loss occurs with disease progression in HD patients and animal models (Ferrante et al., 1991; Klapstein et al., 2001; Laforet et al., 2001; Spires et al., 2004; Lerner et al., 2012; Marco et al., 2013; Rocher et al., 2016; Wu et al., 2016), as well as in DIV21 YAC128 MSNs co-cultured with cortical neurons at a 1:3 cortico:striatal (CS) ratio (Wu et al., 2016; Schmidt et al., 2018). Recently, DAPK1 was found to contribute to cortical spine and synapse degeneration after ischemia *in vivo*, and genetic deletion of the DAPK1 catalytic domain reversed hippocampal spine and synapse loss in Tg2576-APP Alzheimer disease mice (Pei et al., 2015; Shu et al., 2016). Therefore, we sought to determine if inhibiting DAPK1 could prevent or reverse striatal spine loss in YAC128 1:3 CS co-cultures. Consistent with previous reports, YAC128 MSNs co-cultured with cortical neurons at a 1:3 ratio exhibited significantly reduced numbers of total spines at DIV21, and this was primarily due to a loss of mature mushroom-shaped spines with a small contribution of immature (stubby, thin, and filopodia) spine types (Figure 6A). Treatment with DKI from DIV14-DIV21 completely rescued total, mature, and immature spine numbers in YAC128 MSNs to WT control levels (Figure 6A).

Our previous work showed that YAC128 MSN spine instability in co-culture is apparent by DIV18, but not DIV14, meaning DKI treatment from DIV14 to 21 prevented spine instability from occurring. Next, we sought to determine if DKI treatment could reverse the YAC128 spine loss after it had already occurred. To assess this, we performed a late-intervention treatment from DIV18 to 21 and observed complete reversal of total spine numbers, including the near-complete rescue of mushroom spine density with DKI treatment (Supplementary Figure 5). We did not observe a basal genotypic difference in immature spine numbers in this batch of control-treated neurons (Supplementary Figure 5), indicating that this phenotype may show more culture-to-culture variation compared to the reliable and robust loss of mushroom spines observed in all experiments.

Next, we reasoned that if the beneficial effect of inhibiting DAPK1 on spine stability is mediated through its downregulation of exGluN2B function, then blockade of exNMDARs or GluN2B should have the same effect. Similar to DAPK1 inhibition, blockade of exNMDARs with low-dose memantine for 7 days completely rescued total and mature spine numbers in co-cultured YAC128 MSNs (Figure 6B). Surprisingly, GluN2B-selective antagonism with ifenprodil provided no benefit in YAC128 MSNs and significantly reduced mushroom spine density in WT MSNs (Figure 6C), suggesting that ifenprodil may inhibit the beneficial activity of GluN2B-containing synaptic NMDARs in this *in vitro* system. Ultimately, these findings support our hypothesis that DAPK1 activity and consequent upregulation of exNMDAR function contribute to mHTT-induced instability of MSN dendritic spines.

## Discussion

In this study, we provide evidence that DAPK1 plays a key role in regulating the fragile balance between NMDA receptor function and dysfunction and identify DAPK1 as a potential novel therapeutic target for early synaptic protection in HD. DAPK1 is upregulated and activated early in YAC128 brains, contributes to aberrant exGluN2B phosphorylation and surface expression, and promotes dendritic spine loss in cultured MSNs. Our findings support a model whereby at early disease stages, a positive feedback loop between exGluN2B and DAPK1 is amplified in the presence of mHTT, causing exGluN2B surface expression and DAPK1 activity to augment each other. Ultimately, these findings illuminate the exciting potential to mitigate early synaptic dysfunction in HD by therapeutically targeting the activity of DAPK1.

Here, we show that dysregulation of DAPK1 occurs as early as 1 month of age in affected regions of the YAC128 mouse brain, before the emergence of neuropathology or behavioral deficits. This finding mirrors previous reports of NMDA receptor dysfunction and excitotoxicity in YAC128 mice at young ages (Graham et al., 2009; Joshi et al., 2009; Raymond et al., 2011). The increase in DAPK1 protein expression and activation was associated with enhanced exGluN2B binding and phosphorylation at the DAPK1 site, S1303. Despite elevated protein levels, we did not observe altered *Dapk1* mRNA expression in the YAC128 brain compared to WT, suggesting stabilization or reduced turnover of the protein. This phenomenon has previously been observed for DAPK1 in both Alzheimer’s and Parkinson’s disease mouse models (Kim et al., 2014; Su et al., 2019). To support this hypothesis, there is evidence that both activation (S308 dephosphorylation) of DAPK1 and phosphorylation of GluN2B at S1303 lead to a stronger GluN2B-DAPK1 interaction (Goodell et al., 2017), which could presumably incorporate DAPK1 into NMDAR complexes, rendering it less accessible to degradation by proteasomes (Jin et al., 2006). Thus, elevated DAPK1 levels may be secondary to the activation of the protein. Our pulse-chase experiments support this by showing that inhibition of DAPK1 kinase activity with a selective inhibitor results in more rapid DAPK1 protein turnover *in vitro*.

In the present study, *in vivo* blockade of exNMDARs with low-dose memantine rescued aberrant DAPK1 pS308 levels as well as exGluN2B pS1303 in the cortex of YAC128 mice. This suggests that activation of DAPK1 and phosphorylation of S1303 are downstream consequences (either direct or indirect) of exNMDAR activity. Previously, memantine treatment in YAC128 mice normalized non-PSD GluN2B expression, indicating a direct correlation between activity and expression of the receptor in the extrasynaptic compartment (Dau et al., 2014). Plausibly, the mechanism of this regulation could involve activity-induced DAPK1-mediated phosphorylation of GluN2B at S1303. To support this theory, we have shown that small-molecule inhibition of DAPK1 reduces GluN2B S1303 phosphorylation in the YAC128 model, both *in vitro* and *in vivo*, and that DAPK1 inhibition, mutation of the DAPK1 active site (K42), or mutation of the GluN2B S1303 residue significantly reduce GluN2B surface expression. Of note, we found that a single intranasal dose of a selective DAPK1 inhibitor (TC-DAPK6) decreased exGluN2B pS1303 levels in cortical tissue from 4-week-old YAC128 mice. This was associated with a partial reduction in the level of exGluN2B itself. Altogether, these results suggest that an amplified positive feedback loop involving exGluN2B S1303 phosphorylation and DAPK1 activity may contribute to GluN2B dysfunction in the extrasynaptic compartment in HD. We propose that decreasing pS1303 with the DAPK1 inhibitor may lead to reduced exGluN2B surface expression *via* receptor internalization, thus targeting these receptors for degradation and ultimately normalizing pathologically elevated exGluN2B levels themselves. To date, only one other report has described the delivery of TC-DAPK6 to the rodent brain *in vivo*. In that case, the compound was microinjected directly into the cortex, and biochemical alterations were observed 2 days post-treatment, while antidepressant effects persisted at 7 days (Li et al., 2018). For chronic neurodegeneration, an ideal treatment will be non-invasive and allow for repeated dosing, which may be essential to produce a long-term therapeutic benefit. The present work demonstrates that it is possible to deliver TC-DAPK6 to the brain by intranasal administration, thus providing the opportunity to conduct minimally-invasive repeated-dosing studies.

DAPK1 and CaMKII compete with each other for binding to GluN2B (Goodell et al., 2017), and phosphorylation of GluN2B at S1303 negatively regulates its interaction with CaMKII (Strack et al., 2000; Raveendran et al., 2009). When bound, CaMKII anchors casein kinase 2 (CK2) at receptor complexes, allowing it to phosphorylate GluN2B at S1480 (Sanz-Clemente et al., 2013). This disrupts the postsynaptic density protein 95 (PSD95)-GluN2B interaction and induces clathrin-mediated receptor endocytosis (Nakazawa et al., 2001; Lavezzari et al., 2003; Chung et al., 2004; Prybylowski et al., 2005; Sanz-Clemente et al., 2013). Therefore, phosphorylation of exGluN2B at S1303 by DAPK1 may increase exGluN2B function by unlinking CaMKII and indirectly promoting PSD95-dependent stabilization of GluN2B at the cell surface. An increase in the PSD95-GluN2B interaction has been previously reported in extrasynaptic striatal fractions from YAC128 mice and was shown to contribute to the elevated GluN2B surface expression consistently observed in this model (Fan et al., 2009).

Age-associated dendritic spine loss occurs in multiple mouse models of HD as a consequence of synaptic dysfunction and CS disconnect (Graveland et al., 1985; Ferrante et al., 1991; Spires et al., 2004; Lerner et al., 2012; Marco et al., 2013; Buren et al., 2016; Rocher et al., 2016; Wu et al., 2016). Our optimized 1:3 CS neuronal co-culture is a “chronic” *in vitro* model which naturally develops many HD-like synaptic phenotypes over 3 weeks and is therefore advantageous over traditionally-used models of NMDAR dysfunction which rely on acute toxic insult (i.e., glutamate or NMDA) to induce rapid cell death (Schmidt et al., 2018). We observed complete protection against spine instability in co-cultured YAC128 MSNs treated with a DAPK1 inhibitor. Notably, mature mushroom spines were preserved, suggesting the maintenance of functional activity, although this was not directly assessed. Low-dose memantine treatment had a similar beneficial effect on spine structure, suggesting that both compounds may act in the same pathway. Importantly, when we treated DIV18 cultures with the DAPK1 inhibitor, we successfully reversed spine instability after it had already occurred. Thus, although DAPK1 is dysregulated early in the YAC128 mouse model, it is may still be an attractive target for late intervention therapies aimed at restoring the synaptic function of surviving neurons. Our results highlight the dynamic nature of mHTT-induced spine instability and support the hypothesis that it may be possible to reverse spine pathology after it has emerged by inhibiting the activity of DAPK1.

The mechanism by which DAPK1 inhibition prevents spine instability remains to be further explored. Here, we propose three possible pathways that could be involved in mediating this effect. First, Ca^2+^ influx through NMDARs can induce further inositol triphosphate receptor (IP3R)- or ryanodine receptor (RyR)-mediated Ca^2+^ release from the ER which, if occurring in excess, results in mitochondria depolarization and neuronal death (Ruiz et al., 2009; Ferreira et al., 2015). Depleted ER Ca^2+^ stores in YAC128 neurons also causes enhanced store-operated Ca^2+^ entry (SOCE) and consequent spine degeneration (Wu et al., 2016). It remains to be determined if ER depletion is caused by an upstream increase in exGluN2B activity. Plausibly, potentiation of exGluN2B Ca^2+^ currents by DAPK1 could result in greater Ca^2+^ release from ER stores, thus promoting SOCE and spine loss in HD. Second, ERK can be sequestered in the cytosol by DAPK1 (Chen et al., 2005; Xiong et al., 2018), which may prevent its activation and subsequent activity toward the CREB-kinase ribosomal s6 kinase (Chen et al., 2005; Roskoski, 2012). DAPK1 lowering may thus release ERK to indirectly promote CREB phosphorylation and another pro-survival signaling. Importantly, both ERK1/2 and CREB are activated by synaptic NMDAR activity and dominantly shut off by extrasynaptic NMDARs (Hardingham et al., 2002; Ivanov et al., 2006). In presymptomatic YAC128 mice (1–2 months of age), both CREB and ERK1/2 activation are impaired (Milnerwood et al., 2010; Gladding et al., 2014). Therefore, activated DAPK1 in 1-month-old YAC128 mice could provide a link between elevated exGluN2B activity and CREB shut-off *via* ERK1/2, which would be expected to impact the stability of spines and synapses. Finally, CaMKII itself also directly mediates CREB S133 phosphorylation (Sun et al., 1994; Yan et al., 2016). Since DAPK1 overexpression inhibits synaptic CaMKII localization and interaction with GluN2B (Goodell et al., 2017), it is possible that DAPK1 lowering could influence CREB activity and consequent synaptic stabilization mechanisms by enhancing GluN2B/CaMKII-mediated signaling.

ExNMDAR hyperactivity plays a critical role in early synaptic dysfunction and phenotype onset in HD, making it an ideal highly-upstream target for neuroprotection (Milnerwood et al., 2010, 2012; Dau et al., 2014). GluN2B forms a key component of the NMDAR signaling hub, interacting with over 70 cytosolic and membrane proteins to influence neuronal activity and function (Lu et al., 2018). Furthermore, the subunit is subjected to post-translational processes which may be exploited to modify its function (Lussier et al., 2015). Despite the complexity of GluN2B regulation, its highly unique relationship with DAPK1 provides the opportunity for an innovative therapeutic approach. The present work supports DAPK1 as a novel therapeutic target for synaptic protection in HD as well as other major neurodegenerative disorders involving heightened exNMDAR activity. Furthermore, these results warrant the testing of chronic DAPK1 inhibition *in vivo* for the development of novel therapeutics, as well as a deeper investigation into the precise mechanisms underlying the contribution of DAPK1 to synaptic dysfunction.

## Data Availability Statement

The raw data supporting the conclusions of this article will be made available by the authors, without undue reservation.

## Ethics Statement

The animal study was reviewed and approved by University of British Columbia Animal Care Committee.

## Author Contributions

MS designed, performed, and analyzed all Western blotting, subcellular fractionation, primary neuronal culture, and imaging experiments, prepared all of the figures, and wrote the manuscript. NC, AA, and MS designed and performed *in vivo* drug treatments and subsequent interpretation of results. FL and MS designed, performed, and analyzed *in vitro* pulse-chase evaluation of DAPK1 turnover and generated figures. NC, AA, and FL provided comprehensive intellectual input and edited the manuscript. LD assisted with the optimization and execution of Western blotting, subcellular fractionation, co-immunoprecipitation, cell culture, tissue collection, and sample processing. NL, BN, and LA assisted with the optimization and execution of cell culture, transfection, co-immunoprecipitation, imaging, and pulse-chase experiments. YK assisted with *in vivo* drug treatments and animal monitoring. LR and MH supervised the project, provided intellectual input, and edited the manuscript. All authors contributed to the article and approved the submitted version.

## Conflict of Interest

MH was an employee of Teva Pharmaceuticals, Inc. during part of this study. Teva did not play a role in the design, collection, analysis, interpretation of data, or funding of this study.

The remaining authors declare that the research was conducted in the absence of any commercial or financial relationships that could be construed as a potential conflict of interest.

## Supplementary Material

SUPPLEMENTARY FIGURE 1 Western blot validation of subcellular fractionation methods. **(A)** A standard subcellular fractionation protocol leads to enrichment of the synaptic proteins PSD95 and GluN2B in the synaptic (post-synaptic density, PSD) fraction, P, while the presynaptic marker synaptophysin (SYP) is enriched in the extrasynaptic (non-PSD) compartment, NP. **(B)** A modified “non-synaptic,” NS, lysis protocol using a gentle buffer followed by harsh lysis of the remaining insoluble synaptic pellet, P, (i.e., with buffer containing SDS and sodium deoxycholate) leads to a similar distribution profile for PSD95 and GluN2B, as observed in panel **(A)**. SYP was not enriched in the NS fraction, potentially due to the inclusion of cytosolic protein in this lysate which could dilute any observable enrichment of presynaptic proteins.

SUPPLEMENTARY FIGURE 2 DAPK1 protein expression and activation are unaltered in the YAC18 cortex. Cortical tissues from 1-month-old WT and YAC18 (Y18; line 212) were lysed in a stringent buffer and total lysate was run by SDS-PAGE and Western blot for DAPK1, pS308, and β-actin. Data are normalized to WT values (*n* = 8 biological replicates, two technical replicates each; Student’s *t*-test).

SUPPLEMENTARY FIGURE 3 DAPK1 protein expression is elevated in both synaptic and extrasynaptic membrane fractions in the 1-month YAC128 brain. **(A)** Cortical or **(B)** striatal tissues were subjected to subcellular fractionation to isolate synaptic (PSD) and extrasynaptic (non-PSD) membranes, followed by Western blotting for DAPK1 expression. Data are normalized to WT values (*n* = 8–20 biological replicates; Student’s *t*-test, **p* < 0.05,***p* < 0.01).

SUPPLEMENTARY FIGURE 4 CaMKII protein expression and autonomous activation are unaltered in the 1-month YAC128 brain. Cortical or striatal tissues were lysed for **(A,D)** total protein or subjected to subcellular fractionation to isolate **(B,E)** synaptic (PSD) or **(C,F)** extrasynaptic (non-PSD) membranes. Samples were processed by SDS-PAGE and Western blotting for CaMKII expression and phosphorylation at the autonomous activation site T286. Data are normalized to WT values and presented as mean ± SEM (*n* = 8 biological replicates, two technical replicates each; student’s *t*-test).

SUPPLEMENTARY FIGURE 5 DAPK1 inhibition reverses spine instability in YAC128 MSNs. WT and YAC128 1:3 CS co-cultures were treated from DIV18–21 with DMSO (0.01%) or the DAPK1 inhibitor TC-DAPK6 (DKI, 1 μM), and spine analysis was performed on DARPP32+ MSNs. **(A)** Representative dendritic images from each condition. **(B)** Total WT and YAC128 MSN spine density at DIV18. **(C)** Total, mushroom, and immature spine density at DIV21 after 3 days of treatment with either DMSO or DKI. Data are presented as absolute values (*n* = 24 cells from three independent cultures; two-way ANOVA with Bonferroni *post hoc* analysis, ***p* < 0.01, ****p* < 0.001). Scale bar = 5 μm.

## Abbreviations

AHA: azidohomoalanine
AP5: amino-5-phosphonovaleric acid; a competitive NMDA receptor antagonist
BACHD: Transgenic mouse model of Huntington disease containing the full-length human mutant huntingtin gene with 97 mixed CAA-CAG repeats
BDNF: brain-derived neurotrophic factor
C6R: Transgenic mouse model expressing full-length human mutant huntingtin bearing a point mutation at the D586 caspase cleavage site
CaMKII: calcium/calmodulin-dependent protein kinase II
CB: cerebellum
CK2: casein kinase 2
COS-7: fibroblast-like cell line derived from monkey kidney tissue
CREB: cAMP response element-binding protein
CS: cortico-striatal
CTX: cortex; cortical
DAPK1: death-associated protein kinase 1
DARPP32: dopamine- and cAMP-regulated neuronal phosphoprotein
DIV: day-*in vitro*
DKI: DAPK1 inhibitor; also referred to as TC-DAPK6
DMSO: dimethyl sulfoxide
ER: endoplasmic reticulum
ERK1/2: extracellular signal-regulated kinase 1/2
exGluN2B: extrasynaptic GluN2B-containing NMDAR
exNMDAR: extrasynaptic NMDAR
FLAG: polypeptide protein tag (sequence DYKDDDK)
FVB: wild-type inbred laboratory mouse strain
GFP: green fluorescent protein
GluN2B: NMDA receptor subunit type 2B
HTT: huntingtin
IFEN: ifenprodil
IP3R: inositol triphosphate receptor
JNK: c-Jun N-terminal kinase
MAPK: mitogen-activated protein kinase
MEM: memantine
mHTT: mutant huntingtin
MSN: medium spiny neuron
NMDA: N-methyl D-aspartate
NMDAR: N-methyl D-aspartate receptor
nNOS: neuronal nitric oxide synthase
PSD: postsynaptic density
PSD95: postsynaptic density protein 95
RT: room temperature
RyR: ryanodine receptor
SOCE: store-operated calcium entry
STR: striatum; striatal
TC-DAPK6: DAPK1 inhibitor; also referred to as DKI
VGLUT1: vesicular glutamate transporter 1
WT: wild-type
YAC128: Transgenic mouse model of Huntington disease containing the full-length human mutant huntingtin gene encoding 125 glutamines
YAC18: Transgenic mouse model containing the full-length human wild-type huntingtin gene encoding 18 glutamines
YFP: yellow fluorescent protein.
