# Supplementary figures and images for: DAPK1 Promotes Extrasynaptic GluN2B Phosphorylation and Striatal Spine Instability in the YAC128 Mouse Model of Huntington Disease
Source: Front Cell Neurosci. 2020 Nov 5;14:590569. doi: 10.3389/fncel.2020.590569 (PMC7674490; doi:10.3389/fncel.2020.590569)

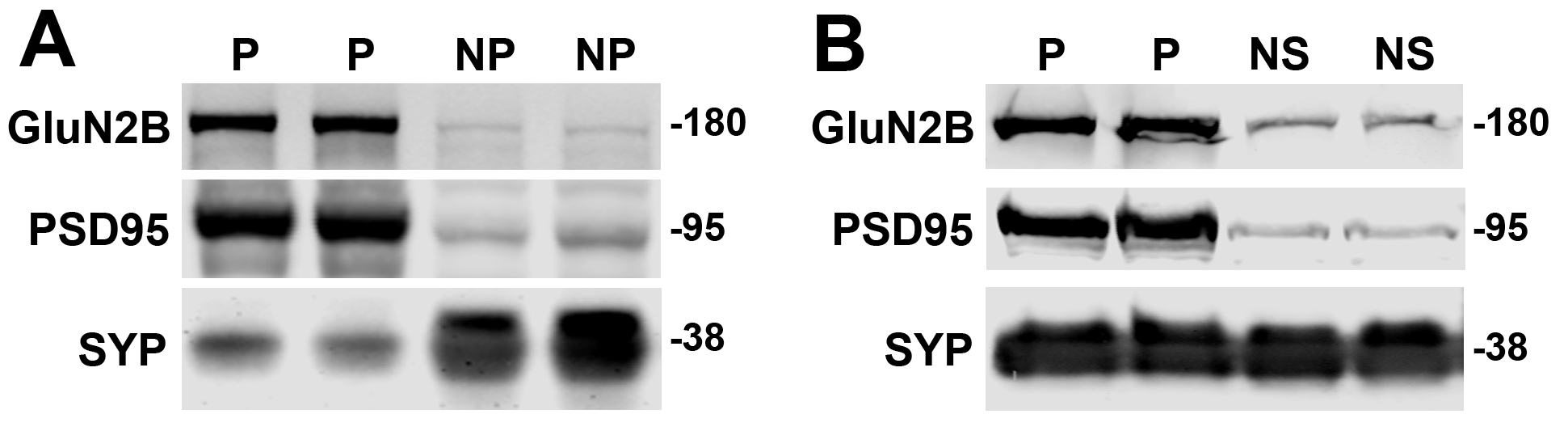

Supplement: SUPPLEMENTARY FIGURE 1 — Western blot validation of subcellular fractionation methods. (A) A standard subcellular fractionation protocol leads to enrichment of the synaptic proteins PSD95 and GluN2B in the synaptic (post-synaptic density, PSD) fraction, P, while the presynaptic marker synaptophysin (SYP) is enriched in the extrasynaptic (non-PSD) compartment, NP. (B) A modified “non-synaptic,” NS, lysis protocol using a gentle buffer followed by harsh lysis of the remaining insoluble synaptic pellet, P, (i.e., with buffer containing SDS and sodium deoxycholate) leads to a similar distribution profile for PSD95 and GluN2B, as observed in panel (A). SYP was not enriched in the NS fraction, potentially due to the inclusion of cytosolic protein in this lysate which could dilute any observable enrichment of presynaptic proteins. [file Image_1.jpeg]

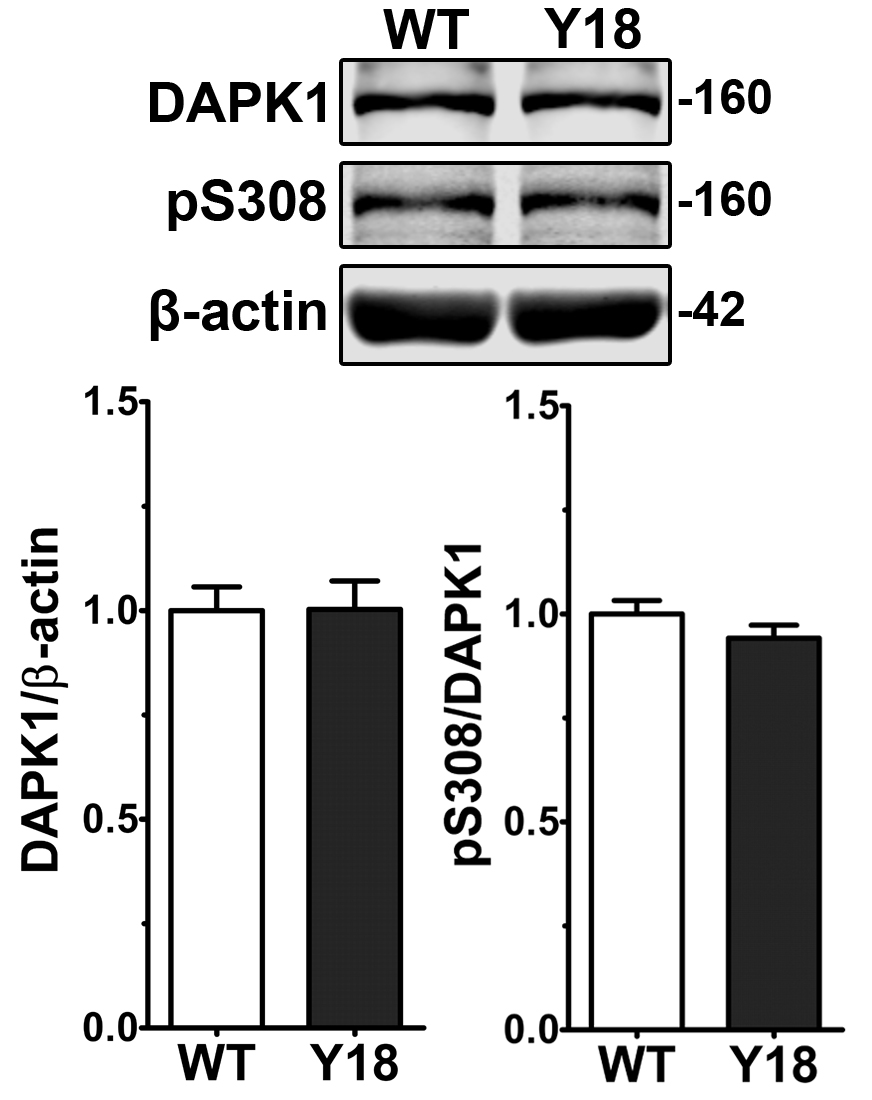

Supplement: SUPPLEMENTARY FIGURE 2 — DAPK1 protein expression and activation are unaltered in the YAC18 cortex. Cortical tissues from 1-month-old WT and YAC18 (Y18; line 212) were lysed in a stringent buffer and total lysate was run by SDS-PAGE and Western blot for DAPK1, pS308, and β-actin. Data are normalized to WT values (n = 8 biological replicates, two technical replicates each; Student’s t-test). [file Image_2.jpeg]

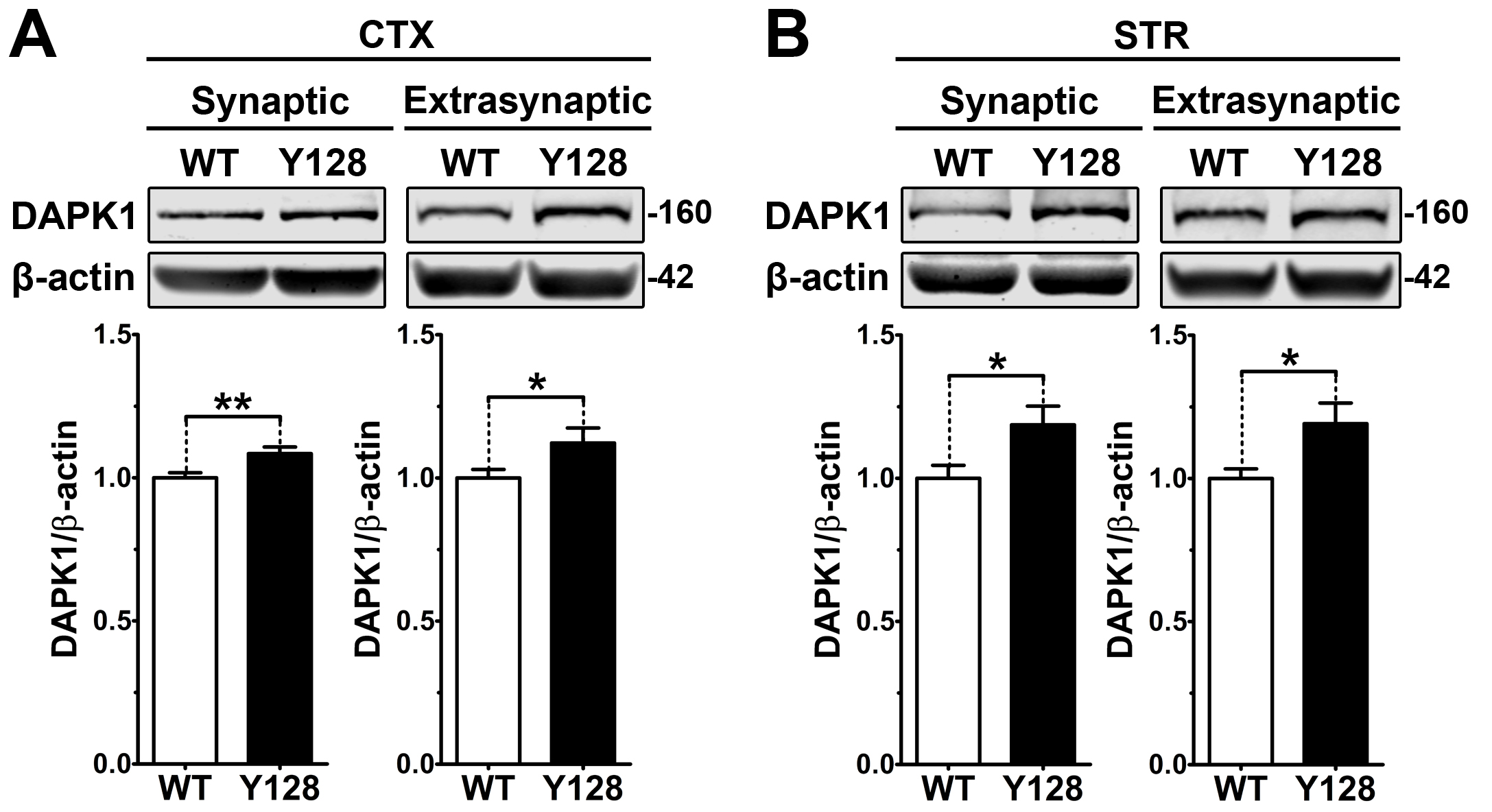

Supplement: SUPPLEMENTARY FIGURE 3 — DAPK1 protein expression is elevated in both synaptic and extrasynaptic membrane fractions in the 1-month YAC128 brain. (A) Cortical or (B) striatal tissues were subjected to subcellular fractionation to isolate synaptic (PSD) and extrasynaptic (non-PSD) membranes, followed by Western blotting for DAPK1 expression. Data are normalized to WT values (n = 8–20 biological replicates; Student’s t-test, *p < 0.05,**p < 0.01). [file Image_3.jpeg]

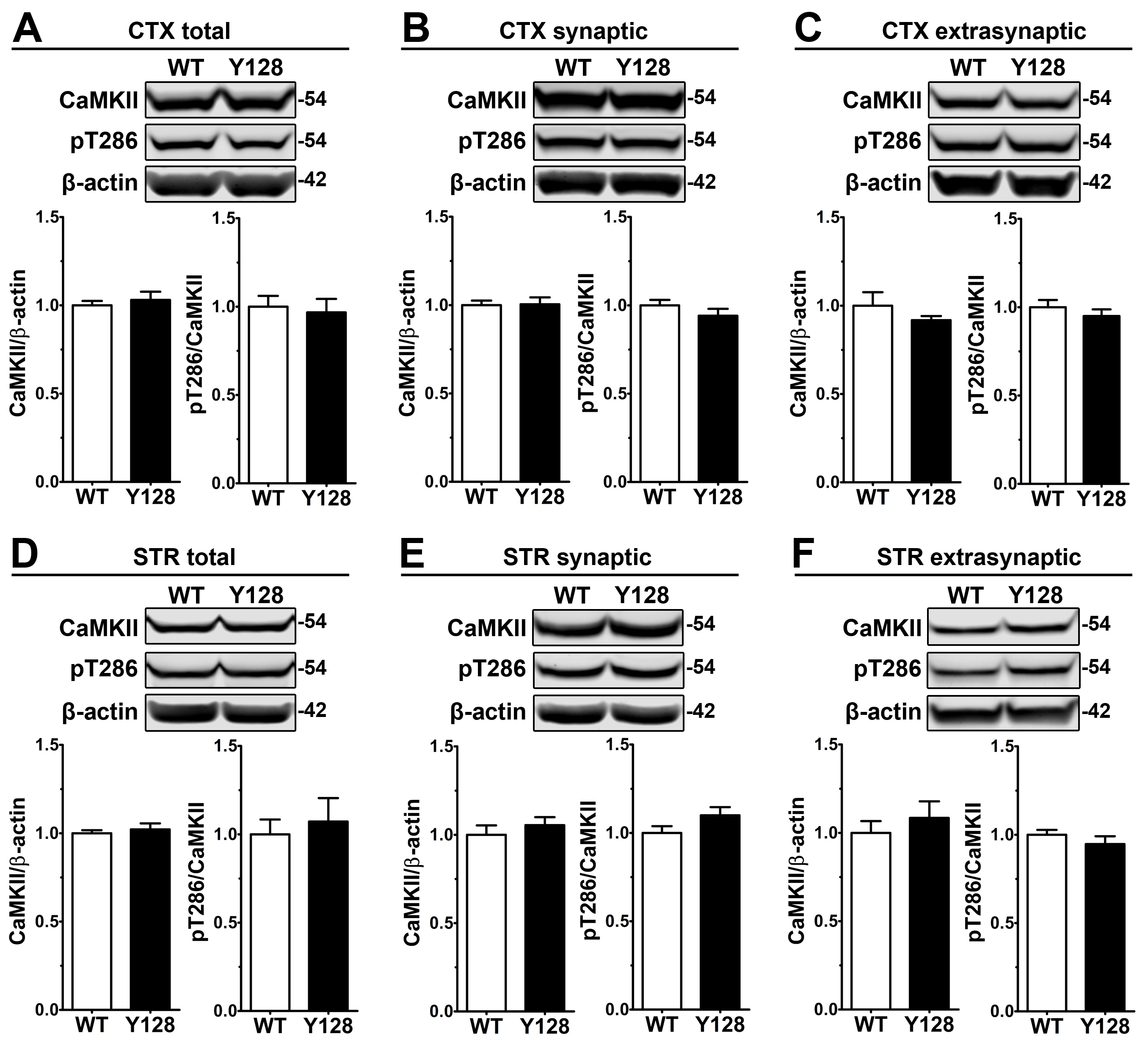

Supplement: SUPPLEMENTARY FIGURE 4 — CaMKII protein expression and autonomous activation are unaltered in the 1-month YAC128 brain. Cortical or striatal tissues were lysed for (A,D) total protein or subjected to subcellular fractionation to isolate (B,E) synaptic (PSD) or (C,F) extrasynaptic (non-PSD) membranes. Samples were processed by SDS-PAGE and Western blotting for CaMKII expression and phosphorylation at the autonomous activation site T286. Data are normalized to WT values and presented as mean ± SEM (n = 8 biological replicates, two technical replicates each; student’s t-test). [file Image_4.jpeg]

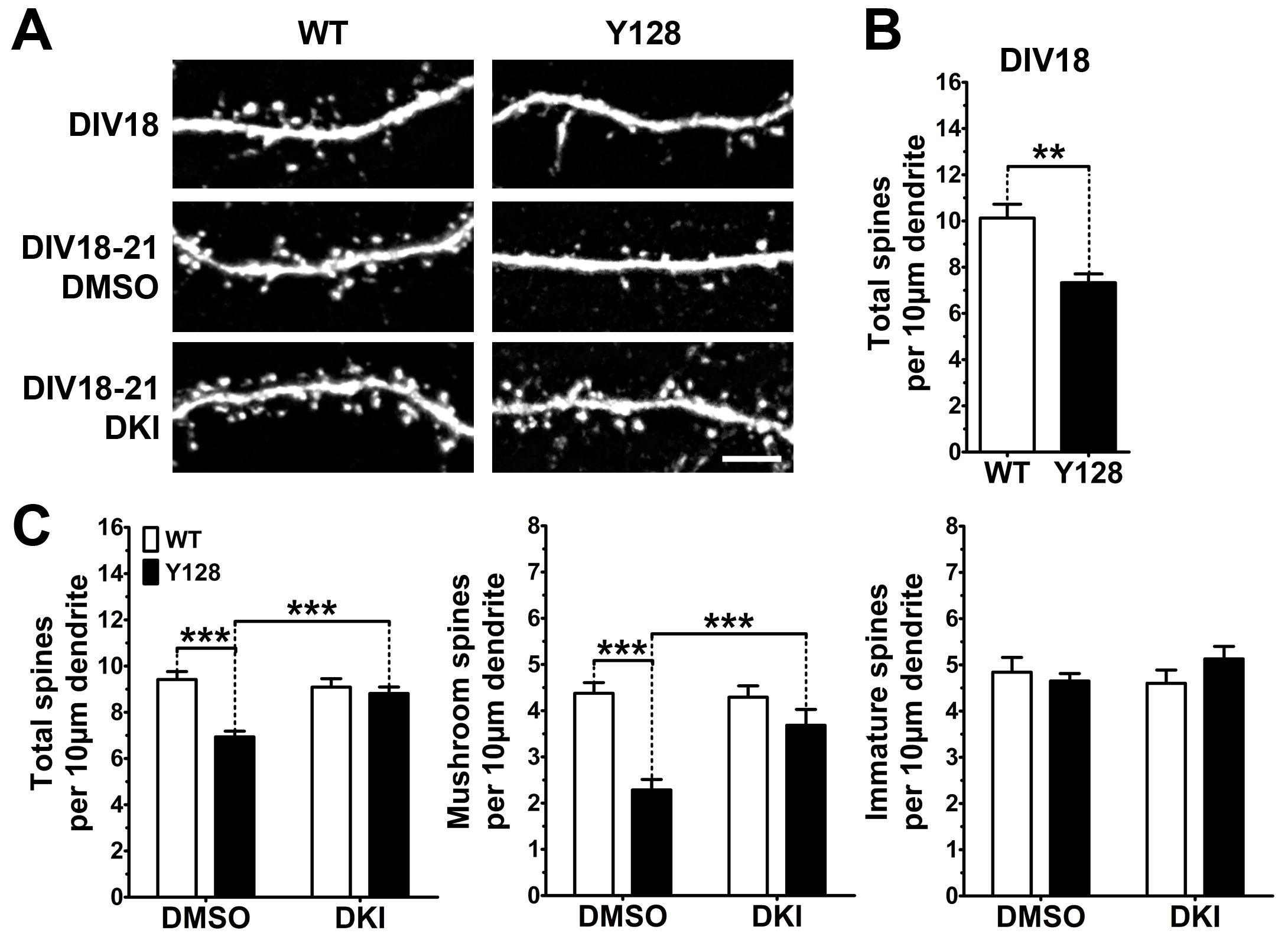

Supplement: SUPPLEMENTARY FIGURE 5 — DAPK1 inhibition reverses spine instability in YAC128 MSNs. WT and YAC128 1:3 CS co-cultures were treated from DIV18–21 with DMSO (0.01%) or the DAPK1 inhibitor TC-DAPK6 (DKI, 1 μM), and spine analysis was performed on DARPP32+ MSNs. (A) Representative dendritic images from each condition. (B) Total WT and YAC128 MSN spine density at DIV18. (C) Total, mushroom, and immature spine density at DIV21 after 3 days of treatment with either DMSO or DKI. Data are presented as absolute values (n = 24 cells from three independent cultures; two-way ANOVA with Bonferroni post hoc analysis, **p < 0.01, ***p < 0.001). Scale bar = 5 μm. [file Image_5.jpeg]
